# Cell‐specific differences in mitochondrial calcium signaling in Alzheimer's disease

**DOI:** 10.1002/alz70855_101755

**Published:** 2025-12-23

**Authors:** Shatakshi Shukla, Ashlesha Kadam, Shanikumar Goyani, Dhanendra Tomar, Pooja Jadiya

**Affiliations:** ^1^ Wake Forest University School of Medicine, Winston Salem, NC, USA; ^2^ Wake Forest University School of Medicine, Winston‐Salem, NC, USA

## Abstract

**Background:**

We and others have demonstrated that mitochondrial dysfunction, particularly excessive mitochondrial calcium (_m_Ca^2+^) accumulation in neurons, serves as an early hallmark of Alzheimer's disease (AD), driving neuronal cell death and metabolic dysregulation. While genetic modulation of _m_Ca^2+^ uptake and efflux in neurons shows promise in mitigating mitochondrial dysfunction. However, AD pathology involves disrupted coordination among neurons, microglia, and astrocytes. These cell‐specific mechanisms remain poorly understood. Our study addresses this critical gap by investigating cell‐type‐specific differences in _m_Ca^2+^ flux and signaling under normal and AD conditions.

**Method:**

To model AD, we generated stable human neuronal (SH‐SY5Y), microglia (HMC3) and astrocyte (SVGp12) cell‐line harboring AD‐linked APP mutations (Swedish, Florida and London; APPswe/F/L). We analyzed the expression of the mitochondrial calcium uniporter (mtCU) using qRT‐PCR and assessed _m_Ca^2+^ influx and efflux using ratiometric Ca^2+^ reporter Fura‐FF. We measured mitochondrial calcium retention capacity (CRC) to evaluate the Mitochondrial Permeability Transition Pore (MPTP) opening. Additional assays measured ATP, NADH, cell death, mitochondrial structure, membrane potential and mitochondrial ROS using a combination of imaging and biochemical approaches. Mitochondria isolated by sucrose‐density gradient centrifugation were analyzed for lactylation, a novel post‐translational modification, via western blotting.

**Result:**

Our results reveal that mitochondrial dysfunction occurs in all brain cells, with neurons showing the highest susceptibility to cell death and reduced bioenergetics. Astrocytes and microglia exhibit elevated _m_Ca^2+^ signaling, higher uptake rates, and greater calcium retention capacity compared to neurons, reflecting cell‐specific roles in energy demand and stress responses. Notably, cell‐specific differences in lactylation were observed, with significantly higher expression in microglia and astrocytes, suggesting a role in mitochondrial functional regulation.

**Conclusion:**

Cell‐specific _m_Ca^2+^ signaling dynamics influence the metabolic and pathophysiological contributions of brain cells to AD. Identifying the regulatory mechanisms underlying _m_Ca^2+^ signaling dysregulation in each cell type offers potential therapeutic targets to restore cellular communication and function. Our findings provide insights into mitochondrial bioenergetics, cell death pathways, and AD progression, paving the way for novel therapeutic strategies targeting brain cell‐specific mechanisms.

